# Establishment of Orthotopic Liver Tumors by Surgical Intrahepatic Tumor Injection in Mice with Underlying Non-Alcoholic Fatty Liver Disease

**DOI:** 10.3390/mps1020021

**Published:** 2018-06-04

**Authors:** Zachary J. Brown, Bernd Heinrich, Tim F. Greten

**Affiliations:** Gastrointestinal Malignancy Section, Thoracic and Gastrointestinal Oncology Branch, Center for Cancer Research, National Cancer Institute, National Institutes of Health, Bethesda, MD 20892, USA; bernd.heinrich@nih.go (B.H.); tim.greten@nih.gov (T.F.G.)

**Keywords:** hepatocellular carcinoma, orthotopic mouse model, immunotherapy, bioluminescent imaging

## Abstract

The prevalence of non-alcoholic fatty liver disease (NAFLD) and its advanced form, nonalcoholic steatohepatitis (NASH), is increasing, and as such its contribution to the development of hepatocellular carcinoma is also rising. NAFLD has been shown to influence the immune tumor microenvironment. Therefore, development of pre-clinical mouse models in the context of NAFLD are increasingly important. Here, we describe a mouse model designed to recapitulate the findings of NAFLD followed by rapid induction of orthotopic liver tumors with intrahepatic tumor injection. Additionally, we utilized bioluminescent imaging to monitor tumor growth and response to therapy. The development of one dominant tumor nodule allows precise separation of tumor and liver tissue. This is useful for immunotherapy studies as mononuclear cells from the tumor and the surrounding liver tissue can be analyzed separately.

## 1. Introduction

Liver cancer is a common cause of cancer-related death worldwide with approximately 810,000 deaths in 2015 [1]. Systemic treatment options for hepatocellular carcinoma (HCC) are limited [2]. As such, improved pre-clinical studies are required to develop novel therapies. Non-alcoholic fatty liver disease (NAFLD) and its advanced form, nonalcoholic steatohepatitis (NASH), are recognized as the liver disease associated with metabolic syndrome and characterized by increased fat deposition in the hepatocytes [3,4]. The prevalence of NAFLD is increasing rapidly with the growing epidemics of diabetes and obesity. NAFLD is present in up to one-third of the general population and the majority of patients with obesity or type 2 diabetes. Not only does NAFLD carry the health risks associated with metabolic syndrome but it also increases the risk of developing HCC which is the most common primary liver cancer in adults [5]. Among those affected by NAFLD, the incidence of HCC is 0.44 per 1000 person-year and that incidence is increased to 5.29 per 1000 person-year for NASH [3].

Our group has shown that NAFLD causes a selective loss of CD4^+^ T cells initiated by excessive mitochondrial intake of fatty acids [6,7]. Therefore, in order to effectively study the effects of fatty liver disease on immune-based therapies, we designed a model of intrahepatic tumor injection in combination with diets that promote NAFLD. The methionine- and choline-deficient (MCD) diet is able to induce hepatic steatohepatitis in approximately 10 days but the mice fail to develop accompanying insulin resistance and obesity, and in fact the mice lose weight [8,9,10,11]. Similarly, the choline-deficient l-amino-defined (CDAA) diet results in liver fibrosis, but takes longer than the MCD diet to induce liver disease (approximately 22 weeks). Like the MCD diet, the CDAA also fails to recapitulate the accompanying symptoms of obesity and insulin resistance [12,13,14]. Conversely, the high-fat diet (HFD) produces obesity and insulin resistance with hepatic fibrosis but takes approximately nine weeks to develop liver disease [9]. Here, we present an in vivo mouse model protocol to induce HCC on the background of fatty liver disease in a relatively short experimental timeframe. Although the protocol of intrahepatic injection has been described in other models [15], this protocol is designed to recapitulate the findings of fatty liver disease followed by rapid induction of HCC with intrahepatic tumor injection. Even though the MCD diet does not fully recapitulate all aspects of metabolic syndrome, its rapid onset of NAFLD makes it more ideal to study with an intrahepatic injection of tumor cells. The intrahepatic tumor injection may also be utilized in mice with a normal liver (no liver disease) to provide a control to the effects rendered by NAFLD. Additionally, we utilized bioluminescent imaging (BLI) to monitor tumor growth and response to therapy.

## 2. Experimental Design

**Figure 1:**
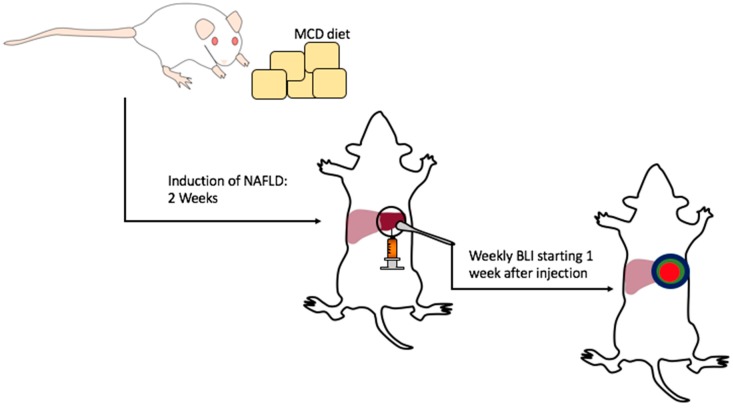
This protocol describes a preclinical mouse model of orthotopic hepatocellular carcinoma (HCC) development in mice with underlying non-alcoholic fatty liver disease (NAFLD). NAFLD is established by feeding mice the methionine- and choline-deficient (MCD) diet for two weeks. After two weeks on the MCD diet, orthotopic liver tumors are established by a surgical liver injection of tumor cells and bioluminescent imaging (BLI) is performed one week after tumor establishment.

This protocol describes a preclinical mouse model of orthotopic HCC development in mice with underlying NAFLD by implantation of syngeneic HCC cell lines which can be monitored by BLI (Figure 1). This model can be used to study treatment strategies and also cell biology and immunology/immunotherapy in the setting of HCC with underlying NAFLD/NASH. The final readout can be achieved in a reasonable amount of time, within 4–5 weeks, after starting the experiment. For our purpose, we utilized the luciferase expressing HCC cell line RIL-175 [16]. Expression of the luciferase (LUC) gene in RIL-175 synthesizes the enzyme luciferase which allows for monitoring tumor progression during a course of treatment. This technique uses the detection of luminescence emitted after administration and oxidation of the substrate luciferin [17]. However, it has also been demonstrated that when tumors reach the end stage of disease, BLI may underestimate actual tumor burden although tumor growth may be progressing on other imaging modalities such as MRI [18,19]. We have also utilized this protocol with different HCC and non-HCC cell lines, such as the B16 melanoma cell line, with successful results.

Our model was utilized to study immunotherapy and as a result we could not take advantage of immunocompromised nude mice which are commonly used for BLI imaging [19,20]. Therefore, to achieve the best results of monitoring tumor growth, we recommend utilizing mice with white fur as the darker fur can influence the sensitivity of BLI [20]. The RIL-175 cell line we used is derived from C57BL/6 mice [16], therefore we utilized B6(Cg)-Tyr^c-2J^/J mice (B6-albino stock #000058) purchased from The Jackson Laboratory (Bar Harbor, ME, USA). All animals in these studies were housed and experiments were performed according to the institutional guidelines and approved by a NCI-Bethesda (Bethesda, MD, USA) Institutional Animal Care and Use protocol. Animal well-being was observed on a daily basis.

### 2.1. Materials

- Phosphate-buffered saline (PBS) without Ca/Mg (ThermoFisher, Grand Island, NY, USA; Cat. no.: 14190-144)
- Cell culture medium, RPMI (ThermoFisher; Cat. no.: 61870-036)
- Fetal bovine serum (FBS; Gemini Bio-Products, West Sacramento, CA, USA; Cat. no.: 100-106)
- Glutamate (ThermoFisher; Cat. no.: 25030081)
- Penicillin/streptomycin antibiotics (ThermoFisher; Cat. no.: 15140-122)
- Trypsin (Sigma-Aldrich, St. Louis, MO, USA; Cat. no.: 59427C)
- Matrigel (Corning, Corning, NY, USA; Cat. no.: 354230)
- 70% ethanol (Sigma-Aldrich; Cat. no.: 459836-2L)
- Povidone iodine 10% solution (Qualitest, Huntsville, AL, USA; Cat. no.: NDC 0603-1550-58)
- Methionine- and choline- deficient (MCD) diet (Research Diets, New Brunswick, NJ, USA; Cat. no.: A02082002B)
- Luciferase-expressing cell line: RIL-175 [16].
- Vicryl suture (Ethicon, Blue Ash, OH, USA; Cat. no.: J391H)
- G (0.33 mm) × 12.7 mm BD Insulin Syringe (BD Bioscience, Franklin Lakes, NJ, USA; Cat. no.: 324702)
- Serological pipettes and pipette tips
- TC Flask T75 (Sarstedt, Newton, NC, USA; Cat. no.: 83.3911)
- Serological pipettes and pipette tips (Sarstedt)
- Eppendorf and centrifuge tubes tubes (Sarstedt)
- Cotton-tipped applicators (Medline, Northfield, IL, USA; Cat. no.: MDS202000)

### 2.2. Equipment

- Incubator
- Centrifuge
- Counter top vortex
- Isoflurane (gas anesthesia system)
- Electric clipper
- Bead sterilizer
- Heating pad
- Heat lamp
- Straight forceps
- Straight scissor
- Needle driver
- Xenogen in vivo imaging system (IVIS Spectrum, Caliper Live Sciences, Hopkinton, MA, USA)

## 3. Procedure

### 3.1. Induction of NAFLD/NASH. Time for Completion: 2 Weeks

**Figure 2:**
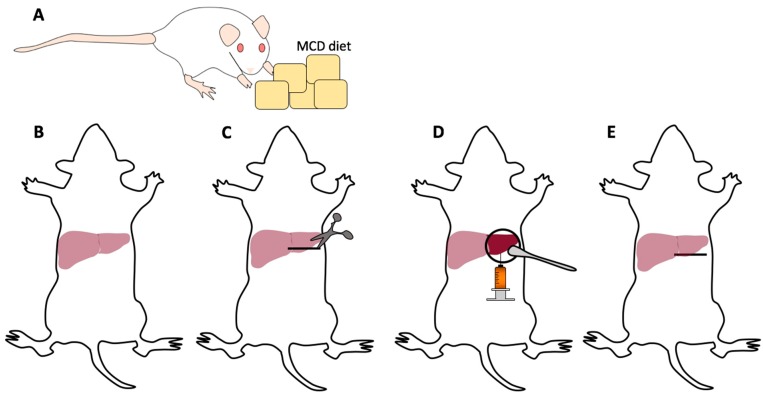
Flow scheme of intrahepatic injections. (**A**) Mice are fed the MCD diet to induce liver disease for approximately two weeks. (**B**) A mouse is anesthetized with 2% isoflurane in the anesthesia chamber, shaved, and placed supine in the anesthesia nose cone on a heating pad. The abdomen is then disinfected for the procedure. (**C**) An approximately 1 cm horizontal incision is made over the left upper abdomen. (**D**) The liver is withdrawn and stabilized with a cotton-tipped applicator and a subcapsular injection of the tumor suspension is performed applying gentle pressure to ensure hemostasis. (**E**) The liver is returned into the abdomen which is then sutured closed in two layers. Post-operative anesthetic is administered, and the mouse is allowed to recover.

**Figure 3:**
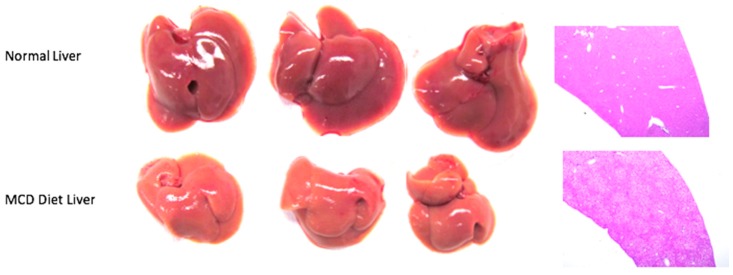
Resulting liver disease due to MCD diet.

1. Mice should be started on the MCD diet at approximately 6–8-weeks-old for two weeks for the induction of NAFLD/NASH (Figure 2A).
  NOTE: Mice on MCD may lose up to 30% of their body weight while on the MCD diet and as such weight should be monitored periodically during the course of the experiment. Any mouse with severe weight loss should be sacrificed in accordance with institutional protocol.
2. Intrahepatic tumor injections should take place approximately two weeks after induction of the MCD diet when the mice are approximately 8–10-weeks-old. The investigator can visualize the liver disease as discolored often shrunken livers with steatosis on hematoxylin and eosin (H & E) staining (Figure 3).
  NOTE: Although the full extent of liver disease may not be realized after two weeks on diet, this allows for in parallel development of liver disease and HCC which mimics the human condition.
  NOTE: In general, older mice are easier to inject as the liver capsule is often more developed. We advise avoiding intrahepatic injection in mice <8 weeks old.

### 3.2. Cell Preparation. Time for Completion: 2 Weeks

1. Prepare a single cell suspension of a luciferase expressing cell line. Luciferase-expressing RIL-175 HCC tumor cells were cultured in complete RPMI medium supplemented with 10% FBS. Cells should be in culture for approximately four passages prior to injection.
2. Prepare cell injection solution. For tumor establishment, 20 µL of tumor cell suspension should be injected per mouse. Cells should be counted and suspended 10^5^–10^6^ cells per 20 µL in a 50:50 solution of PBS and Matrigel. The concentration of the cell in the cell suspension may vary depending on growth kinetics of the cells being injected.
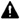
**CRITICAL STEP** Cell suspension and insulin syringes should be kept on ice to prevent Matrigel from solidifying. Stock of Matrigel should be aliquoted during first thaw and divided up into Eppendorf tubes of desired volume and stored at −20 °C.
  NOTE: Matrigel is used for tumor injection to provide a matrix plug allowing tumor cells to remain in liver after injection [15]. Injection of tumor cells in PBS has been performed but Matrigel allows for more consistent results.

### 3.3. Surgical Procedure. Time for Completion: 15–30 Min per Mouse

1. Anesthetize the mice using 2% isoflurane in an anesthesia chamber.
2. Once mice are anesthetized, shave the abdomen clearing the surgical field of fur.
3. Place and keep the mice on a heating pad with anesthesia nose cone to maintain sedation (Figure 2B).
4. Use 10% povidone iodine solution followed by 70% ethanol to disinfect the skin, repeating this process three times.
5. Sterilize the tips of surgical instruments in a bead sterilizer.
6. Grab the skin with forceps and make an approximately 1 cm horizontal incision from the midline toward the left upper abdomen (Figure 2C).
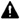
**CRITICAL STEP** Proper placement of the incision makes the procedure much easier. Improper placement may lead to a struggle delivering the liver out of the wound for injection. We recommend making the incision just below the left costal margin but inferior enough not to cut into the thoracic cavity.
  NOTE: Mice on MCD diet have diseased liver and maybe smaller and retracted into the upper abdomen making the incision placement critical.
7. Gently free the skin from the peritoneum. Grab the peritoneum lifting straight upward and make a small incision. Extend the peritoneal incision horizontally following the skin incision ensuring to see the tip of the scissors as to not injure underlying organs.
  NOTE: Bleeding may occur from superficial vessels of the abdominal wall. Use gentle pressure or the application of silver nitrate to help control the bleeding. Blood will distort the field of view and make following steps more difficult.
8. Insert a cotton-tipped applicator into the left upper abdomen under the left lobe of the liver. Remove the cotton-tipped applicator slowly and gently rotate it toward yourself (Figure 2D).
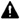
**CRITICAL STEP** We aim to inject the left lobe of the murine liver as it is generally easily accessible and larger than the right or middle lobes. Additionally, the left lobe better accommodates the injected volume without tearing the liver capsule.
  NOTE: The tumor cell suspension should be loaded into the syringe prior to extraction of the liver and kept on ice.
9. With gentle traction stabilize the left lobe of the liver with the left hand against the underlying cotton-tipped applicator.
10. Insert the 29 G needle into the liver parenchyma traversing several millimeters and slowly inject the tumor suspension.
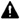
**CRITICAL STEP** Proper needle location for injection is vital for consistent and reproducible results. The needle should traverse several millimeters of liver to prevent backflow of the tumor suspension. The needle should be superficial and visible through the liver and the tumor suspension should be injected just below the liver capsule, which would raise a wheel/bubble. Avoid excessive superior traction of the needle while in the liver parenchyma to prevent lacerating the liver.
  NOTE: This procedure takes surgical skill and practice to perfect in order to obtain reproducible results. It should be noted the amount of time it takes individual investigators to perform the procedure as you do not want the tumor cells to be on ice for an extended period of time. From our experience, after approximately 2–3 h, tumor cells lose viability while on ice.
11. After the injection, slowly retract the needle from the liver and place gentle pressure on the needle insertion site with a cotton-tipped applicator for several minutes to stop bleeding.
12. Using a 5-0 Vicryl suture, close the peritoneal layer with a single figure-of-eight or two single interrupted sutures (Figure 2E).
13. Using a 5-0 Vicryl suture, close the skin with two figure-of-eight or multiple single interrupted sutures.
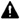
**CRITICAL STEP** Do not use a running suture to close the skin. Mice will bite the sutures. If one suture is used and the mouse bites it, the entire wound may open.
  NOTE: Clips may be used if BLI imaging is not planned on being performed. If following tumor growth with BLI is planned, clips may cause interference with the imaging.
14. Apply post-operative analgesia as per institutional protocol.
15. Recover the mice from anesthesia in a cage with a heat lamp.
16. Closely monitor the mice post-operatively for signs of distress. In combination with MCD diet, mice with intrahepatic tumors generally survive three weeks post-injection (five weeks after the initiation of MCD diet) after which the mice should be sacrificed for humane endpoints.
  NOTE: Upon sacrifice of mice, tumor growth throughout the abdomen is likely the results of a failed intrahepatic injection.

### 3.4. Tumor Growth Monitoring by Bioluminescent Imaging. Time for Completion: 15 Min per Group of Mice

1. Tumor growth is monitored on a weekly basis with BLI.
2. Anesthetize the mice using 2% Isoflurane in an anesthesia chamber.
3. Shave the abdomen to decrease the interference with BLI.
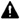
**CRITICAL STEP** From our experience, monitoring intrahepatic injections with BLI in mice with black fur gives unreliable results as the black fur produces increased background signal. Therefore, we utilized B6(Cg)-Tyr^c-2J^/J mice to reduce the background signal. Shave the mice prior to every imaging session to ensure consistent results.
4. Administer an intraperitoneal injection of 150 mg/kg of D-luciferin in PBS.
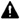
**CRITICAL STEP** Wait for the tumor saturation plateau to be reached. For our RIL-175 cells this was at approximately eight minutes after injection.
5. Perform imaging. We utilized the Xenogen in vivo imaging system The CCD camera was cooled to between −105 °C and −120 °C and the field of view set to 25 cm. Images were acquired with an exposure time of 30 s, medium binning, 1.2 f/stop, with an open filter.
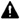
**CRITICAL STEP** Prior to starting a large experiment, it is important to determine the proper exposure time required as to not saturate the image.
6. Recover the mice from anesthesia in a cage with a heat lamp.

## 4. Expected Results

**Figure 4:**
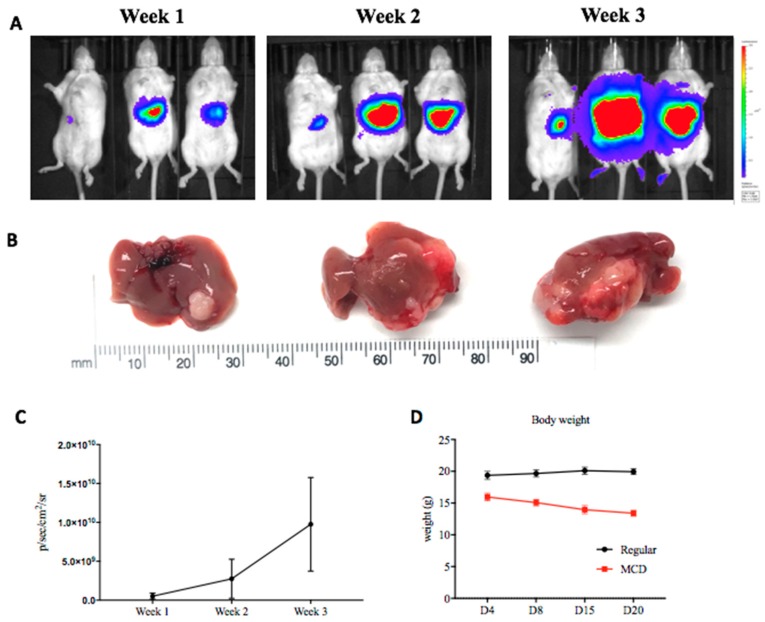
(**A**) IVIS imaging results after intrahepatic tumor injection. (**B**) Resulting tumors after the sacrifice of mice monitored by BLI. (**C**) Cumulative tumor growth was monitored by BLI and reported in photons/s/cm^2^/steradian (p/s/cm^2^/sr). (**D**) Change in the body weight of mice over the course of an experiment. Day 0 is when the intrahepatic tumor injection is performed. The MCD diet was initiated two weeks prior to the establishment of intrahepatic tumors.

The protocol described here outlines a technique utilized for rapid induction of orthotopic liver tumors in mice with concurrent NAFLD. Researchers are starting to recognize the importance of the tumor microenvironment (TME) on HCC development, progression, and response to therapy [6,7,21]. As our group demonstrated NAFLD influences the TME through selective loss of CD4^+^ T cells [6,7], this technique is useful in studying the influences of this finding on different immune based therapies. Additionally, BLI is a useful tool to monitor changes in tumor growth between different treatment modalities. Figure 4A–C demonstrate increased tumor growth over the course of an experiment as measured by BLI and confirmed after the mice were sacrificed.

When obtaining images with BLI, several factors should be kept in mind including mouse fur, the depth of tumor in the tissue, and imaging parameters [20]. These are important considerations as imaging parameters may change depending on the tumor cell line, and the location of the tumor, i.e., subcutaneous versus intrahepatic. Furthermore, during the course of an experiment, mouse body weight should be regularly monitored as mice on the MCD diet often lose weight [10]. Figure 4D depicts changes in mouse body weight over the course of an experiment. Note how mice on the MCD diet have a lower starting body weight four days after the establishment of intrahepatic tumors (diet was initiated two weeks prior to the injection) with a gradual decrease in body weight. If severe weight loss is recognized (>20% starting body weight), the mouse should be sacrificed for humane endpoints as per institutional protocol, and therefore the experiment should be designed accordingly. Additionally, the investigator must keep the weight loss in mind if planning to use this model for a long-term experiment.

**Figure 5:**
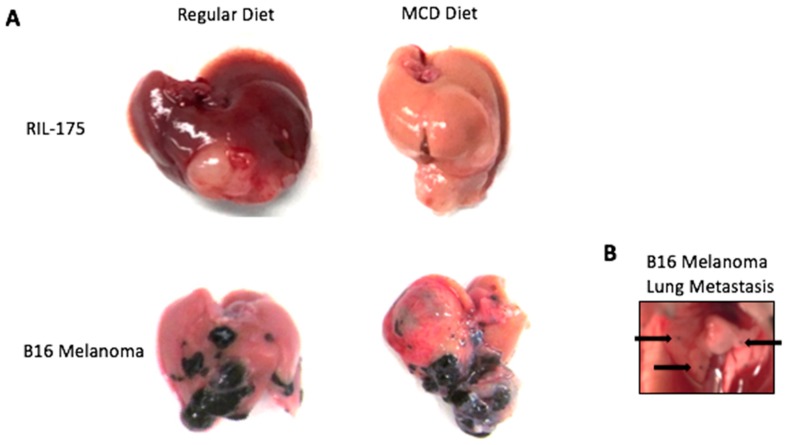
(**A**) Resulting RIL-175 HCC tumors and B16 melanoma tumors three weeks after intrahepatic injection in normal non-diseased (regular diet) and NAFLD (MCD diet) livers. (**B**) Lung metastasis were noted in mice injected with B16 melanoma cells (arrows).

Due to the local injection of tumor cells, mice develop a solid single tumor nodule. Figure 5 displays the results of RIL-175 HCC cells and B16 melanoma cells injected into both normal non-diseased liver and NAFLD liver as the result of the MCD diet. Additionally, in later stages of disease, the mice may develop local metastases in adjacent lobes of the liver as well as lung metastases can be observed (Figure 5).

Development of one big tumor nodule allows for the precise separation of tumor and liver tissue. This is in contrast to other models such as genetically engineered mice which produce several tumors throughout the liver [6]. Having one dominant tumor nodule is useful for immunotherapy studies as mononuclear cells from the tumor and surrounding liver tissue can be analyzed separately. This protocol is a surgical procedure on living mice and there is a learning curve to acquire the skills required to effectively and safely perform the procedure. Basic suturing and safe surgical techniques should be acquired before performing this procedure. Nevertheless, this protocol provides a useful tool to study orthotopic liver tumors in the context of NAFLD.
